# Effects of duodenal transection timing on clinical short-term outcomes of patients with laparoscopic spleen-preserving splenic hilar lymphadenectomy for advanced proximal gastric cancer

**DOI:** 10.1186/s12957-019-1590-z

**Published:** 2019-03-14

**Authors:** Zifang Zheng, Limin Wu, Chenxing Jian, Yucheng Song, Wei Liu

**Affiliations:** Department of Minimally Invasive Surgery, The Affiliated Hospital of Putian University, 999 Dongzhen East Rd, Putian, 351100 Fujian China

**Keywords:** Gastric cancer, Proximal gastric cancer, Duodenal transection timing, Laparoscopy, Laparoscopic-assisted total gastrectomy, Lymphadenectomy

## Abstract

**Background:**

To determine the optimal timing of duodenal transection in patients undergoing laparoscopic-assisted total gastrectomy (LATG) in combination with laparoscopic spleen-preserving splenic hilar lymphadenectomy (LSPL) for advanced proximal gastric cancer (APGC).

**Methods:**

One hundred twenty-seven patients with APGC who received LATG with duodenal transection as well as LSPL between January 2017 and July 2018 were retrospectively recruited in this study. According to the different transection timing, the patients were allocated into two groups: a conventional group (CG) who received the duodenal transection prior to the LSPL and an experimental group (EG) who were given LSPL before the duodenum was transected. Clinical short-term outcomes were compared in the two groups.

**Results:**

Analysis of the demographical and clinical characteristics showed that the two groups were comparable with no significant differences between CG and EG in the study patients regardless of their body mass indices (BMI). The intraoperative and postoperative indicators for clinical short-term outcomes were compared between the CG and EC, and results indicated that the EG had significant shorter mean time of LSPL and total operation time than those in the CG (*P* < 0.05). Of note, the numbers of patients with intraoperative injury and the volume of blood loss during the LSPL procedure were significantly reduced in the EG versus CG (*P* < 0.05). For the obese APGC patients, administration of LSPL prior to duodenal transection significantly increased the number of dissected No.10 lymph nodes (LNs) (*P* < 0.05). The other intraoperative and postoperative indicators did not show any differences between the two comparison groups.

**Conclusions:**

Our findings demonstrated that duodenal transection timing was significantly associated with clinical short-term outcomes of APGC patients. The duodenal transection prior to the LSPL is superior overall to the conventional transection timing in the treatment of APGC patients with LATG and LSPL in combination.

## Introduction

Gastric cancer (GC) is the third leading cause of cancer-associated death worldwide. Moreover, the incidence of proximal gastric cancer (PGC) has increased during the past decade [1]. For those PGC cases detected at advanced stages—namely advanced proximal gastric cancer (APGC)—the clinical outcome or the prognosis is generally very poor. Laparoscopic radical gastrectomy is well accepted and is widely used as a safe, feasible, and effective operative procedure for treating advanced PGC [2–4]. D2 radical gastrectomy is currently the standard surgery for APGC.

According to the criteria outlined in the Japanese Gastric Cancer Treatment Guidelines 2014 (ver. 4), the standard D2 lymphadenectomy for APGC includes the splenic hilar lymph node dissection procedure [5]. A reasonable surgical approach and lymph node dissection in that order result in the successful implementation of laparoscopic surgery for the treatment of APGC. Different surgical approaches, including the left approach, right approach, anterior approach, and posterior approach, have been used during laparoscopic total gastrectomy (LTG) [6]. In a previous study of laparoscopic radical resection for distal gastric cancer, the anterior approach was found to be superior to the posterior approach in lymph node dissection of the superior pyloric region [7]. However, it remains unknown whether splenic hilar lymph node dissection could be affected after duodenal transection.

In the present study, we performed a retrospective and cross-sectional analysis of data from a total of 127 APGC patients who underwent laparoscopic-assisted total gastrectomy (LATG) with LSPL and we aimed to investigate the effects of the timing of duodenal transection on the clinical short-term outcomes of these APGC patients. The study findings were expected to provide a potentially better approach and eventually improve our care for patients with APGC.

## Methods

### Patients and study design

A total of 127 patients with APGC who underwent LATG with laparoscopic spleen-preserving splenic hilar lymphadenectomy (LSPL) as well as duodenum transection at the Department of Minimally Invasive Surgery of the Affiliated Hospital of Putian University during the period between January 2017 and July 2018 were retrospectively recruited in this cross-sectional study. The diagnosis of APGC was pathologically confirmed. The detailed inclusion criteria used in this study were as follows: (1) advanced gastric cancer originating in the proximal third of the stomach; (2) APGC with clinical stage of T2-4aN0-2 M0, which was examined based upon the TNM staging system of the 8th edition of the American Joint Committee on Cancer (AJCC) staging manual; (3) PGC patients who received LATG with D2 lymph node dissection as well as LSPL; and (4) pathological examination which gave negative results for the proximal margin.

The APGC patients who had the following conditions were excluded from the analysis: (1) ASA ≥ IV; (2) intraoperative laparoscopic exploration of tumor peritoneal implantation, invasion of adjacent organs, lymphadenopathy, and fusion into a mass close to arteries and veins and which cannot be removed; (3) preoperative neoadjuvant chemotherapy; and (4) history of previous proximal abdominal surgery.

One hundred twenty-seven patients were allocated into two groups based on the timing of the duodenum transection: a conventional group (*n* = 57) who received the duodenum transection prior to the splenic hilar lymph node dissection and an experimental group (*n* = 70) who were given the splenic hilar lymph node dissection before the duodenum was transected. Demographic and clinical characteristics of the study patients in the two groups were analyzed—including gender, age, hierarchical analysis by body mass index (BMI) [8], tumor size, American Society of Anesthesiologists (ASA) classification, T-stage, N-stage, postoperative TNM stage, and tumor differentiation degree.

The study protocol was reviewed and approved by the Medical Ethics Committee of the Affiliated Hospital of Putian University. Due to the retrospective nature of this study, the usual requirement for signed written informed consent forms was waived.

### Laparoscopic total gastrectomy with splenic lymph node dissection

All the study patients had successfully undertaken surgical procedures of LATG in combination with LSPL. Laparoscopically assisted gastric cancer D2 lymph node dissection was performed according to the procedures as previously described in the Japanese Gastric Cancer Treatment Guidelines 2014 (version 4) [5] and Guideline for Laparoscopic Gastrectomy for Gastric Cancer (2016 edition) [9].

Prior to the surgical procedures, all the study patients received general anesthesia with endotracheal intubation and subsequently were placed in the reverse Trendelenburg position. Pneumoperitoneum was established through umbilical puncture. The intra-abdominal pressure (IAP) was maintained between 12 and 15 mmHg, after which a 10-mm trocar port for the laparoscope was inserted below the umbilicus, and a 12-mm trocar port was introduced on the left anterior axillary line 2 cm below the costal margin. Subsequently, a 5-mm trocar port was inserted on the left mid-clavicular line 2 cm above the umbilicus as an accessory port, and a 5-mm trocar port was placed at the contralateral site. A 5-mm trocar was inserted in the right anterior axillary line 2 cm below the costal margin for exposure.

**Fig. 1:**
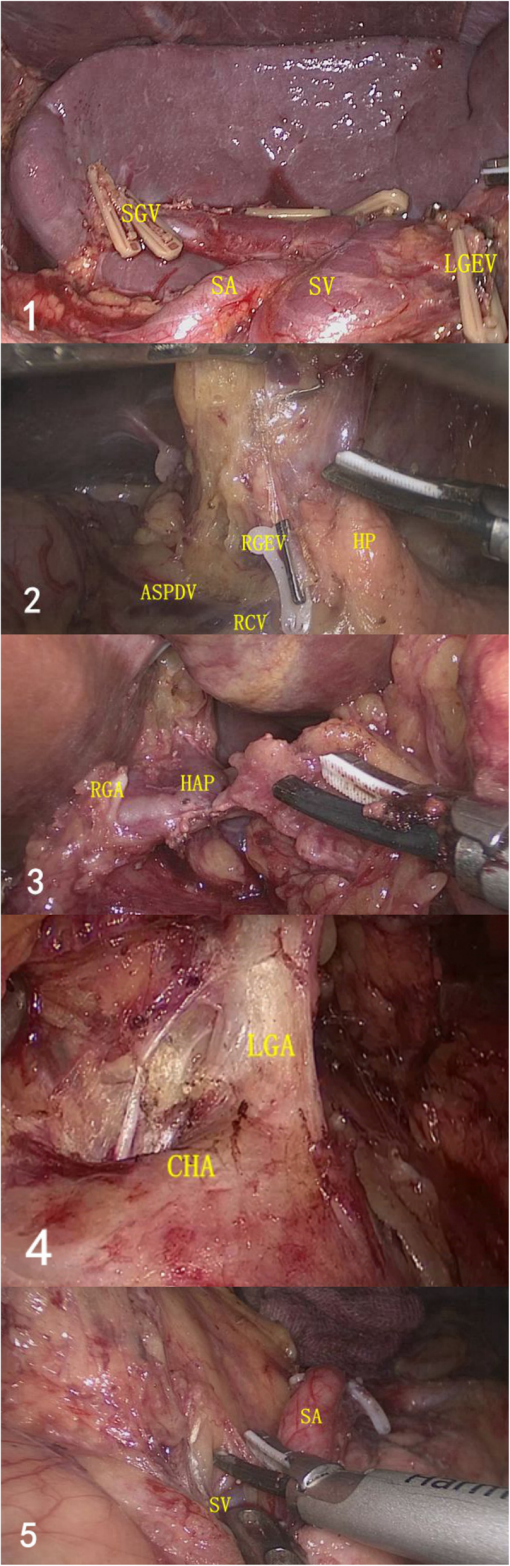
Representative images of laparoscopic total gastrectomy with duodenum transection and splenic lymph node dissection for advanced proximal gastric cancer. **1** Splenic lymph node dissection with “Huang’s three-step method.” **2** Pyloric lymph node dissection. **3** Duodenum was transected prior to dissection of the No.12 lymph nodes **4** Upper pancreatic lymph node was dissected. **5** Dissection of No.11 lymph nodes

For the CG patients, the duodenum was transected before the splenic hilar lymph node dissection procedure. Briefly, the patients in this group were placed with their head elevated approximately 15 to 20° and tilted left-side up approximately 20 to 30°. The gastric colon ligament and the transverse colitis of the transverse mesentery were separated using an ultrasonic knife. Upon completion of the pyloric lymph node dissection, the duodenum was transected and then the upper pancreatic lymph node was dissected. The splenic lymph node dissection was eventually performed using “Huang’s three-step method” [10]. A longitudinal laparotomy was applied, and the specimen was extracted from the peritoneal cavity. The transaction of the esophagus and Roux-en-Y esophagojejunostomy was carried out using a circular stapler. The control group’s radical total gastric D2 lymph node dissection sequence and selecting duodenal transection timing were as follows: No.6→duodenal transection→No.5, 12a, 8a→No.7, 9, 11p→No.3, 1→No.4sb→No.10, 11d→No.2. For the EG patients, the splenic hilar lymph node was dissected before the duodenum was transected. The order of the two procedures was performed differently from the CG. The splenic lymph node dissection based on the aforementioned “Huang’s three-step method” was followed by the pyloric lymph node dissection. As shown in Fig. 1, the duodenum was transected and the upper pancreatic lymph node was dissected. The radical total gastric D2 lymph node dissection sequence and selecting duodenal transection timing in the EG were as follows: No.4sb→No.10, 11d→No.2→No.6→duodenal transection→No.5, 12a, 8a→No.7, 9, 11p→No.3, 1. Each operation was performed at the same department by the same highly experienced surgeon, who led a surgical team and had successfully completed more than 300 laparoscopic gastrectomies for gastric cancer.

### Statistical analyses

Statistical analyses were conducted using SPSS v25.0 statistical software (SPSS Inc., Chicago, IL). Data were expressed as mean ± standard deviation (SD) and were analyzed using the Student *t* test. The detection values were determined based upon the results obtained in the homogeneity of variance test. All numerical data were analyzed and compared with the chi-squared test or Fisher exact test. A *P* value < 0.05 was considered statistically significant.

## Results

### Demographic and clinical characteristics of the study patients

Data were obtained from the 127 APGC patients who successfully undertook LATG with duodenum transection and LSPL. The study patients were divided into the two groups based on the value of body mass index (BMI)—BMI ≥ 24 or BMI < 24—which were further stratified into two subgroups according to the different timing of duodenum transection: CG patients who received the duodenum transection prior to the splenic hilar lymph node dissection and EG patients who were given the splenic hilar lymph node dissection before the duodenum were transected. The demographical and clinical characteristics of all of the study patients are summarized in Tables 1 and 2. Demographical analysis shows that the CG and EG groups were comparable with no significant differences in the age and gender between the two comparison groups regardless of BMI. In addition, there were no significant differences in the clinical characteristics (including tumor size, grade, and other pathological features) between the two comparison groups (CG and EG).

**Table 1:** Demographic and clinicopathological characteristics of the study patients

Characteristics	CG (*n* = 57)	EG (*n* = 70)	*P* value
Age (years)	59.91 ± 10.5	58.71 ± 10.1	0.515
Sex			
Male (*n*)	36	45	0.895
Female (*n*)	21	25	
BMI (kg/m^2^)	21.95 ± 3.0	22.14 ± 2.9	0.715
Tumor size (cm)	3.08 ± 0.6	3.17 ± 0.7	0.456
ASA score (*n*)			
I	22	26	0.977
II	22	27	
III	13	17	
T-stage (*n*)			0.966
T2	13	15	
T3	28	34	
T4a	16	21	
N-stage (*n*)			0.850
N0	10	13	
N1	17	20	
N2	13	20	
N3	17	17	
TNM stage (*n*)			0.369
I	13	13	
II	28	43	
III	16	14	
Histologic grade (*n*)			0.805
Differentiated	24	31	
Undifferentiated	33	39

**Table 2:** Demographic and clinicopathological characteristics of the study patients by BMI

Characteristics	BMI ≥ 24 (kg/m^2^)		*P* value	BMI < 24 (kg/m^2^)		*P* value
	CG (*n* = 18)	EG (*n* = 23)		CG (*n* = 39)	EG (*n* = 47)	
Age (years)	59.5 ± 9.7	57.8 ± 9.6	0.573	60.10 ± 10.9	59.13 ± 10.3	0.673
Sex						
Male (*n*)	11	15	0.786	25	30	0.782
Female (*n*)	7	8		14	17	
BMI (kg/m^2^)	25.81 ± 1.1	25.65 ± 1.0	0.637	20.1 ± 1.5	20.4 ± 1.7	0.496
Tumor size (cm)	3.12 ± 0.8	3.29 ± 0.7	0.449	3.07 ± 0.6	3.11 ± 0.7	0.746
ASA score (*n*)						
I	8	10	1.000	14	16	1.000
II	6	7		16	20	
III	4	6		9	11	
T-stage (*n*)			0.995			0.969
T2	4	5		9	10	
T3	8	10		20	24	
T4a	6	8		10	13	
N-stage (*n*)			0.630			0.713
N0	3	4		7	9	
N1	5	10		12	10	
N2	4	5		9	15	
N3	6	4		11	13	
TNM stage (*n*)			0.593			0.398
I	5	6		8	7	
II	6	11		21	32	
III	7	6		10	8	
Histologic grade (*n*)						
Differentiated	7	10	0.767	17	21	0.919
Undifferentiated	11	13		22	26	

Note: Differentiated: papillary or well/moderately differentiated tubular adenocarcinoma. Undifferentiated: poorly differentiated or mucinous adenocarcinoma or signet-ring cell carcinoma. *P* values indicate comparisons between CG and EG

*Abbreviations*: *CG* conventional group, *EG* experimental group, *ASA* American Society of Anesthesiologists, *BMI* body mass index, *TNM* tumor node metastasis staging, *n* number of patients

### Comparison effects of the timing of duodenum transection on clinical short-term outcomes of the study patients

The intraoperative and postoperative indicators for comparison of the short-term clinical outcomes of the study patients, and resulting data, are presented in Table 3. In the patients with BMI ≥ 24, the mean time of splenic hilar lymph node dissection (LSPL), total operation time, intraoperative injury score, volume of blood loss during LSPL, and number of dissected No.10 lymph nodes were 33.28 min, 247.56 min, 7, 29.83 mL, and 2.50 in the CG group, while in the EC group they were 27.58 min, 232.88 min, 3, 25.13 mL, and 3.25, respectively. The differences between the CG and EC groups (*P* < 0.05) were therefore statistically significant. No significant difference was seen in the remaining indicators, including the mean number of overall dissected lymph nodes, time of postoperative anal exsufflation, time of postoperative fluid diet intake, time of postoperative semi-fluid diet intake, time of intraperitoneal drainage tube removal, postoperative complications, and length of postoperative hospital stay.

**Table 3:** Intraoperative and postoperative indicators of the study patients

Variables	BMI ≥ 24 (kg/m^2^)		*P* value	BMI < 24(kg/m^2^)		*P* value
	CG (*n* = 18)	EG (*n* = 23)		CG (*n* = 39)	EG (*n* = 47)	
SLNs dissection time (min)	33.28 ± 4.0	27.58 ± 3.1	0.000	29.87 ± 3.8	25.21 ± 2.9	0.000
Operation time (min)	247.56 ± 21.9	232.88 ± 18.4	0.023	230.79 ± 17.5	219.74 ± 17.0	0.004
Intraoperative injury (*n*)	7	3	0.047	13	7	0.044
SLNs dissection blood loss (ml)	29.83 ± 5.2	25.13 ± 5.8	0.010	25.67 ± 4.1	21.06 ± 3.8	0.000
Mean retrieved No.10 LNs	2.50 ± 1.1	3.25 ± 1.2	0.047	2.64 ± 1.1	2.81 ± 1.0	0.467
Mean total retrieved LNs	31.0 ± 7.9	32.3 ± 7.2	0.572	30.62 ± 7.2	31.51 ± 6.7	0.553
Time to first flatus (days)	4.28 ± 1.0	4.17 ± 0.7	0.678	3.92 ± 0.9	3.81 ± 0.7	0.503
Time to fluid diet (days)	4.83 ± 0.6	4.75 ± 0.7	0.700	4.3 ± 0.8	4.27 ± 0.9	0.559
Time to soft diet (days)	8.06 ± 0.8	7.83 ± 0.7	0.366	7.51 ± 0.8	7.30 ± 0.9	0.265
Time to drainage tube removal (days)	9.67 ± 0.9	9.54 ± 1.2	0.716	9.38 ± 0.9	9.21 ± 1.0	0.433
Postoperative complications (*n*)	4	4	0.769	6	7	0.950
Postoperative stay(days)	11.56 ± 1.1	10.83 ± 1.2	0.053	10.79 ± 1.1	10.36 ± 1.0	0.064

Note: Intraoperative injury refers only to splenic vascular injury and spleen laceration. *P* values indicate comparisons between the control group and the experimental group

*Abbreviations*: *CG* conventional group, *EG* experimental group, *SLNs* splenic hilar lymph nodes, *n* number of patients

Among the patients with a BMI less than 24, the mean time of LSPL, total operation time, intraoperative injury, and volume of blood loss during LSPL were 29.87 min, 230.79 min, 13, and 25.67 mL, while in the EC group they were 25.21 min, 219.74 min, 7, and 21.06 mL, respectively. The differences were statistically significant between the CG and EC groups (*P* < 0.05), whereas the remaining indicators did not differ (Table 2). The EG patients had a significant shorter mean time of LSPL and total operation time compared to the CG patients (*P* < 0.05). In addition, there were significantly fewer cases of intraoperative injury and volume of blood loss during LSPL decrease in the EG patients versus the CG patients (*P* < 0.05). For the obese APGC patients, more No.10 lymph nodes were retrieved in the EG compared with the CG (*P* < 0.05). These data indicated that the timing of duodenum transection was closely associated with clinical short-term outcomes of the patients, with the EG data being superior (i.e., better patient outcomes) to the CG data. A total of 358 lymph nodes were dissected; the metastasis rate was 10.06% (36/358) of splenic lymph nodes. Among the patients with BMI ≥ 24, 45 lymph nodes were dissected in the control group with a metastasis rate of 8.89% (4/45) while 78 lymph nodes were dissected in the experimental group with a metastasis rate of 10.3% (8/78). Among the patients with BMI < 24, 103 lymph nodes were dissected in the control group with a metastasis rate of 11.65% (12/103) while 132 lymph nodes were dissected in the experimental group with a metastasis rate of 9.09% (12/132).

Anastomotic hemorrhage occurred in one patient who was successfully treated with hemostasis under endoscopy. One of the two patients with abdominal hemorrhage was successfully treated with conservative treatment; the other patient was treated with hemostasis. Complications included abdominal infection, pulmonary infection, inflammatory intestinal obstruction, chylous fistula, and anastomotic leakage. Postoperative complications were graded according to the Clavien-Dindo method, and appropriate treatments were performed [11]; these postoperative complications were all successfully treated using conservative methods. The 30-day mortality rate of the study patients was 0%.

### Postoperative follow-up

The patients were followed up for 3 to 24 months (median 11 months) by telephone, clinic visit, or WeChat (a Chinese messaging and social media app). During the follow-up period, one CG patient and one EG patient developed metastasis and recurrent GC; they were still receiving treatment in our hospital at the time of writing. The remaining patients did not show any clinical signs of recurrence.

## Discussion

Major novel findings in this retrospective study of APGC patients were as follows: (1) the timing of duodenal transection significantly affected clinical short-term outcomes of the APGC patients with LATG and LSPL, (2) administration of LSPL prior to duodenal transection significantly shortened both LSPL and total operation time (*P* < 0.05), (3) administration of LSPL prior to duodenal transection significantly reduced intraoperative injury and the volume of blood loss during the LSPL procedure (*P* < 0.05), and (4) for the obese APGC patients, administration of LSPL prior to duodenal transection significantly increased the number of retrieved No.10 LNs (*P* < 0.05).

Radical surgery has been effective in treating patients with APGC and improving survival times. In 2008, Hyung and colleagues [12] first performed laparoscopic spleen-spleen area lymph node dissection in 15 cases with gastric cancer and achieved satisfactory short-term clinical outcomes to support the safety, feasibility, and effectiveness of these surgical procedures. According to previous reports, the metastatic rate of splenic lymph nodes among patients with APGC was high, ranging from 8.8 to 20.9% [13, 14], for which successfully and completely intraoperative dissection of splenic lymph node has been found to be directly related to the clinical outcomes, such as the postoperative survival time [15].

Patient weight has a significant impact on lymph node dissection [16]. Therefore, subjects were divided into two groups based on BMI stratification. At present, WHO defines a BMI ≥ 25 kg/m^2^ as overweight, which differs from the Chinese Adult Obesity Prevention Expert Consensus 2011 defining ≥ 24 kg/m^2^ as overweight. A BMI of 24 was used as the cutoff value to analyze the difference between the two patient groups.

The deep position of the spleen area, the relatively narrow operating space, the fragile texture of the spleen, and the complex of the vascular anatomy have been challenging for splenic hilar lymph node dissection in D2 lymph node dissection for APGC and are the main limitations. Laparoscopic radical gastrectomy for the treatment of spleen-preserving hilar lymphadenectomy with reduction or minimization of intraoperative bleeding has been demonstrated to be safe and feasible [17].

At present, conventional surgical approaches in China—including the left approach [18], right approach [19], and posterior pancreatic approach [20]—have both advantages and disadvantages. Huang et al. [21, 22] adopted the left approach and summarized a method of laparoscopic spleen-preserving hilar lymph node dissection, terming it “Huang’s three-step method.” With the help of an assistant pulling and exposing, the spleen hilar lymph node was dissected within three steps, which simplified the original complex spleen hilar lymph node dissection, improved the operation efficiency, and reduced the intraoperative complications. Thus, laparoscopic spleen-preserving hilar lymphadenectomy is widely used in our center.

The timing of duodenum transection during the surgical procedures remains debatable. Qian et al. [23] suggested performing the duodenum transection prior to lymph node dissection, as the greater omentum should be blocked by the stomach to enlarge the surgical space, but to maintain appropriate tension in the local operative area. In contrast, Lin et al. [7] recently found that (after lymph node dissection in the subpyloric region) the upper pancreatic lymph node was dissected after the transection of the duodenum, that is, the anterior approach has certain advantages over the posterior approach especially for patients with later stage, higher BMI, and larger tumors. However, it remains unclear whether splenic hilar lymph node dissection could be affected after duodenal transection.

The “Huang’s three-step method” requires assistants to lift, pull, and flip the omentum during the exposure, especially in the second step of lymph node dissection: the assistant needs to connect the omentum between the anterior wall of the stomach and the lower edge of the liver and pull the stomach with the left hand. The bottom of the big curved side is turned to the upper right side [24], and the free omentum is blocked by the stomach to fully expose the surgical field of view. However, these operational procedures are performed in advance of disconnecting the duodenum to form effective tension between the duodenum and the stomach, which enlarges the operative field, in the splenic hilar region, and in turn creates favorable conditions for subsequent lymph node dissection. After the duodenum is transected, it is difficult or impossible for the assistant to perform the operation, especially for patients with later-stage disease, higher BMI, and larger tumors. The space of splenic hilum is narrow, the great curvature of gastric fundus cannot confine the omentum between the liver and stomach, and the free omentum enters the surgical field repeatedly. In addition, the assistant’s lack of experience and skill may affect the thoroughness and safety of lymph node dissection.

In the current study, we found that conducting LSPL prior to duodenal transection significantly reduced both LSPL and total operation time and improved intraoperative injury and the volume of blood loss during the LSPL procedure in the APGC patients regardless of their BMI. Performing LSPL prior to the duodenal transection significantly increased the number of dissected N10 lymph nodes in the APGC patients with BMI ≥ 24 without altering the numbers in the patients with BMI < 24.

LSPL for APGC is difficult and always has a risk of complications, for which we should choose a reasonable surgical approach and lymph node dissection timing. Preoperative three-dimensional CT angiography can be routinely performed to judge the classification of splenic vessels, which can greatly lower difficulty level of surgery, shorten the operation time, and minimize the chances of splenic vascular injury [25]. In addition, good cooperation with each other in a surgical team is important to make the surgery successful and improve the clinical outcomes.

Despite the interesting findings, we also realize that the present study has potential limitations. For instance, this current study was performed using the data on APGC patients obtained from a single hospital. Further prospective randomized controlled trials at multiple hospitals or centers will be needed to further confirm the findings as well as to validate the value of this procedure in laparoscopic-assisted spleen-preserving hilar lymphadenectomy for APGC.

## Conclusion

In conclusion, our results showed that the optimal timing of duodenum transection was significantly associated with clinical short-term outcomes of the APGC patients. The duodenum transection prior to the LSPL is overall superior to the conventional timing in the treatment of APGC patients with LATG in combination with duodenum transection and LSPL.

## Abbreviations

AJCC: American Joint Committee on Cancer
APGC: Advanced proximal gastric cancer
ASA: American Society of Anesthesiologists
ASPDV: Anterior superior pancreaticoduodenal vein
BMI: Body mass index
CHA: Common hepatic artery
GC: Gastric cancer
HAP: Hepatic artery proper
HP: Head of pancreas
IAP: Intra-abdominal pressure
JCGC: Japanese Classification of Gastric Carcinoma
JGCA: Japanese Gastric Cancer Association
LATG: Laparoscopic-assisted total gastrectomy
LGA: Left gastric artery
LGEV: Left gastroepiploic vein
LNs: Lymph nodes
LSPL: Laparoscopic spleen-preserving splenic hilar lymphadenectomy
RGA: Right gastric artery
RGEV: Right gastroepiploic vein
SGV: Short gastric vein
SP: Splenic artery
SV: Splenic vein
